# Long-Term Outcome of Non-Sustained Ventricular Tachycardia in Structurally Normal Hearts

**DOI:** 10.1371/journal.pone.0160181

**Published:** 2016-08-22

**Authors:** Chin-Yu Lin, Shih-Lin Chang, Fa-Po Chung, Yun-Yu Chen, Yenn-Jiang Lin, Li-Wei Lo, Yu-Feng Hu, Ta-Chuan Tuan, Tze-Fan Chao, Jo-Nan Liao, Yao-Ting Chang, Chung-Hsing Lin, Suresh Allamsetty, Rohit Walia, Abigail Louise D. Te, Shinya Yamada, Shuo-Ju Chiang, Hsuan-Ming Tsao, Shih-Ann Chen

**Affiliations:** 1 Division of Cardiology, Department of Medicine, Taipei Veterans General Hospital, Taipei, Taiwan; 2 Department of Medicine, National Yang-Ming University School of Medicine, Taipei, Taiwan; 3 Institute of Epidemiology and Preventive Medicine College of Public Health, National Taiwan University, Taipei, Taiwan; 4 Division of Cardiology, Department of Internal Medicine, School of Medicine, College of Medicine, Taipei Medical University, Taipei, Taiwan; 5 Division of Cardiology, National Yang-Ming University Hospital, Yi-Lan, Taiwan; Scuola Superiore Sant'Anna, ITALY

## Abstract

**Background:**

The impact of non-sustained ventricular tachycardia (NSVT) on the risk of thromboembolic event and clinical outcomes in patients without structural heart disease remains undetermined. This study aimed to evaluate the association between NSVT and clinical outcomes.

**Methods:**

The study population of 5903 patients was culled from the “**R**egistry of 24-hour **E**CG **m**onitoring at **T**aip**e**i Veterans General Hospital” (REMOTE database) between January 1, 2002 and December 31, 2004. Of that total, we enrolled 3767 patients without sustained ventricular tachycardia, structural heart disease, and permanent pacemaker. For purposes of this study, NSVT was defined as 3 or more consecutive beats arising below the atrioventricular node with an RR interval of <600 ms (>100 beats/min) and lasting < 30 seconds.

**Result:**

There were 776 deaths, 2042 hospitalizations for any reason, 638 cardiovascular (CV)-related hospitalizations, 350 ischemic strokes, 409 transient ischemic accident (TIA), 368 new-onset heart failure (HF), and 260 new-onset atrial fibrillation (AF) with a mean follow-up duration of 10 ± 1 years. In multivariate analysis, the presence of NSVT was independently associated with death (hazard ratio [HR]: 1.362, 95% confidence interval [CI]: 1.071–1.731), CV hospitalization (HR: 1.527, 95% CI: 1.171–1.992), ischemic stroke (HR: 1.436, 95% CI: 1.014–2.032), TIA (HR 1.483, 95% CI: 1.069–2.057), and new-onset HF (HR: 1.716, 95% CI: 1.243–2.368). There was no significant association between the presence of NSVT and all-cause hospitalization or new-onset AF.

**Conclusion:**

In patients without structural heart disease, presence of NSVT on 24-hour monitoring was independently associated with death, CV hospitalization, ischemic stroke, TIA, and new onset heart failure.

## Introduction

Non-sustained ventricular tachycardia (NSVT) is a common arrhythmia encountered in modern clinical cardiology. In general, NSVT is defined as 3 or more consecutive ventricular beats with an RR interval of 600 ms and lasting <30 second.[1] It has been ascertained that NSVT episodes can be recorded in a normal population.[2–4] Previous studies have suggested an association between NSVT and increased mortality.[5] However, the prognostic significance of NSVT in structurally normal hearts has not yet been established.[6] During exercise or at the recovery phase, recent clinical studies have shown that NSVT could predict an increase in cardiovascular mortality.[7,8] Yet the prognostic significance in trained athletes was also controversial in previous studies.[9] The role of NSVT in ischemic stroke is less well-studied, but remains a topic of interest to researchers. Previous studies showed the association between ventricular arrhythmia and ischemic stroke.[10,11] These studies suggest that dyssynchronous ventricular contraction might play a role in thromboembolic events. The aim of our study was to evaluate the clinical importance of NSVT in the absence of apparent structural heart disease.

## Methods

### Study Population

This retrospective, observational study was based on the database of “**R**egistry of 24-hour **E**CG **m**onitoring at **T**aip**e**i Veterans General Hospital” (REMOTE).[11] A total of 5903 patients were referred for 24-hour electrocardiography (ECG) monitoring between January 1, 2002 and December 31, 2004 for the following indications: palpitations, syncope, and suspected arrhythmia; hospitalized patients were excluded from the study. The extent of clinical follow-up for each patient was determined by physician decision. Clinical features for these patients, including past medical history, comorbidities, and medications, were obtained from hospital discharge diagnoses, outpatient visits, emergency visits, and the Collaboration Center of Health Information Application (CCHIA), Ministry of Health and Welfare in Taiwan.[11–13] The exclusion criteria in our study were as follows: participants with prevalent sustained ventricular tachycardia, permanent pacemaker, heart failure (HF), previous myocardial infarction, catheter ablation, pulmonary hypertension or hypertrophic cardiomyopathy, and valvular heart disease. The final sample included 3767 patients for analysis. This methodology has been validated in our previous studies.[11–13] Furthermore, the Institutional Review Board at Taipei Veterans General Hospital, Taipei, Taiwan approved this study (VGH-IRB Number: 2013-08- 002AC#1).

### Follow-Up and Event Ascertainment

Patients with regular medication received scheduled follow-up every 1–3 months depending upon their clinical course. Alternatively, those patients without regular medication were followed-up based on physician decision or after a new event as defined in this study. Follow-up data of all participants was retrieved from Taipei Veterans General Hospital, Taiwan National Health Insurance Research Database (NHIRD) and CCHIA.[11–13] The primary endpoint of this study was all-cause mortality. The secondary endpoints were events of all-cause hospitalization, hospitalization for CV-related conditions, new-onset AF (atrial fibrillation), ischemic stroke, transient ischemic attack (TIA), and new-onset HF. Primary and secondary endpoints were investigated in detail based on initial identification through *International Classification of Diseases*, *Ninth Edition* (ICD-9), with reference to diagnostic codes or mention of an endpoint on discharge summary, and the database of the CCHIA and NHIRD which had been previously validated.[11, 13–15] Hospitalization was defined as an overnight stay in a hospital ward. New-onset systolic HF and new-onset AF were identified and validated by echocardiographic result and ECG report. Stroke and TIA were identified by imaging report and diagnosis at the time of discharge. The overall end of follow-up was February 28, 2013. The mortality data and cause of death were further confirmed by linking with the National Death Registry, which has been validated previously.[14]

### Non-sustained ventricular tachycardia assessment

All subjects underwent 24-hour ambulatory Holter monitoring; the details of Holter monitoring used in this study were mentioned in a previous work.[11] NSVT was defined as 3 or more consecutive beats arising below the atrioventricular node, with an RR interval less than 600 ms and lasting less than 30 seconds.

### Determination of Risk Factors

Data were collected based on demographic characteristics (age and sex). Target comorbidities, such as diabetes mellitus, hypertension, chronic kidney disease, liver disease, and thyroid disease were determined by using the *ICD-9* codes derived from patient medical charts and CCHIA at the time of examination. Baseline AF was defined by baseline 12-lead ECG, or Holter monitoring. Medical history with anti-arrhythmic agent (Class I and III anti-arrhythmic drug) and anti-hypertension medication (including beta-blocker, calcium channel blocker, angiotensin converting enzyme inhibitor/angiotensin receptor blocker, diuretics, and alpha-blocker) was confirmed by medical chart review.

### Statistical Analysis

Baseline patient characteristics were reported as means ± standard deviations for continuous variables, and as percentages for categorical variables. Continuous and categorical variables were compared using the Student’s t-test and chi-square test with Yates’ correction, respectively. Time to event survival analysis was conducted using the Kaplan-Meier method using the log-rank test for comparison between groups. P value < 0.05 were considered as significant. A Cox proportional hazards model was applied to determine multivariate predictors of time to adverse event. The full model included all the variables that were considered statistically significant (P < 0.05) in baseline characteristics.

The relative risk for a given end-point associated with NSVT was estimated by calculating the hazard ratio (HR) using a Cox regression hazards model. This model was run with all baseline parameters that significantly differ with a p value < 0.05 between patient with and without NSVT (i.e., age, sex, hypertension, diabetes mellitus, chronic kidney disease, and use of anti-arrhythmic medication). A death prior to secondary endpoints was considered as a competing risk event. The death-adjusted cumulative incidences of each secondary endpoint were calculated using the Fine and Gray method [16]. Calculations of cumulative incidences and Cox models in the competing risk analysis were carried out using the R language. Comparisons between the 2 groups for cause of death were performed using the chi-square test for categorical variables. We utilized a Forest plot to present the HRs of NSVT in different subgroups of patients with individual risk factors. To determine whether inclusion of NSVT in the model improved the predictive power, discrimination tests were performed with the integrated discrimination improvement (IDI).[17, 18] For the old model, traditional markers for the outcome (i.e., age, sex, hypertension, diabetes mellitus, HF, and anti-arrhythmic medication) were used. Analyses were performed using SPSS statistical software, version 20.0.

## Results

### Baseline Characteristics

**Fig 1:**
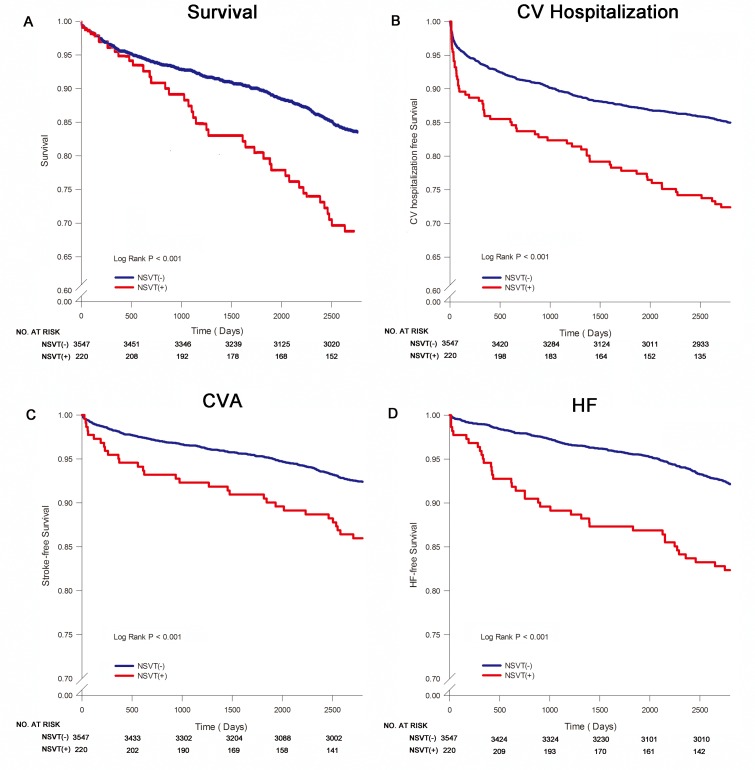
Kaplan-Meier curve of survival by presence of NSVT. Panel A shows Kaplan-Meier survival curve in patients with or without NSVT. Panel B shows Kaplan-Meier curve of CV hospitalization-free survival in patients with or without NSVT. Panel C shows Kaplan-Meier curve of stroke-free survival in patients with or without NSVT. Panel D shows Kaplan-Meier curve of occurrence of new-onset HF free survival in patients with or without NSVT. CV indicates cardiovascular; CVA, cerebral vascular accident; HF, heart failure; NSVT, non-sustained ventricular tachycardia.

The 3767 patients in the study were followed up for 10 ± 1 years. Patients with NSVT were generally older, with a higher prevalence of male, diabetes mellitus, hypertension, and chronic kidney disease, and more anti-hypertensive medications. There were no significant differences in the prevalence of hyperlipidemia, cirrhosis, AF, thyroid dysfunction and pulmonary diseases in these two groups. The baseline data, baseline comorbidities, and patient medications are presented in Table 1. From the total patient study group, 776 (20.6%) patients expired, 2042 (54.2%) were hospitalized, 638 (16.9%) were hospitalized in the cardiology ward, 350 (9.3%) had ischemic stroke, 409 (10.9%) had TIA, 369 (9.8%) were diagnosed as new-onset HF, and 237 (6.3%) were diagnosed with new-onset AF. The results of applying Kaplan-Meier survival curve for different significant endpoints were presented in Fig 1.

**Table 1:** Study Population Characteristics.

Characteristics	NSVT (-)	NSVT (+)	P value
	n = 3547	n = 220	
Age (year)	58. 50±19.60	67.44±15.90	<0.001
Sex (male)	1994(56.2)	164(74.5)	<0.001
Diabetes mellitus	269(7.6)	25(11.4)	0.045
Hypertension	1083(30.5)	97(44.1)	<0.001
Hyperlipidemia	240(6.8)	14(6.4)	0.258
Chronic kidney disease	28(0.8)	8(3.6)	<0.001
Cirrhosis	23(0.6)	0(0.0)	0.231
Atrial fibrillation	238(6.7)	18(8.2)	0.400
Chronic lung disease	98(2.8)	6(2.7)	0.884
Thyroid dysfunction	40(1.1)	2(1.5)	0.843
Sleep apnea	2(0.1)	0(0.0)	0.725
Anti-arrhythmia	13(0.4)	3(1.4)	0.027
Anti-hypertension*	583(16.4)	52(23.6)	0.063
Statin	230(6.5)	13(5.9)	0.213

Values are number of events (%) unless otherwise indicated. NSVT indicates non-sustained ventricular tachycardia

*Beta-blocker, calcium channel blocker, angiotensin converting enzyme inhibitor/angiotensin receptor blocker, diuretics, and alpha-blocker

### NSVT and Long-Term Outcomes

The NSVT group experienced a higher level of mortality, CV hospitalization, stroke, TIA, and new-onset HF compared with the group without NSVT (Fig 1, Table 2, and S1 Table). Cox regression analysis was performed with multivariate adjustment for baseline risk factors (age, sex, hypertension, diabetes mellitus, chronic kidney disease, and anti-arrhythmic medication) for mortality. Further death-adjusted competing risk analysis was performed for all secondary endpoints. The estimated HR (95% confidence interval [CI]) for presence of NSVT remained higher: 1.362 (1.071–1.731, p = 0.012) for all-cause mortality, 1.527 (1.171–1.992, p = 0.002) for CV hospitalization, 1.436 (1.014–2.032, p = 0.041) for new-onset stroke, 1.483 (1.069–2.057, p = 0.018) for new-onset TIA, and 1.716 (1.243–2.368, p = 0.001) for new-onset HF. The estimated HR (95% [CI]) for presence of NSVT was insignificant: 1.175 (0.990–1.395, p = 0.066) for all-cause hospitalization and 1.428 (0.914–2.231, p = 0.118) for new-onset AF.

**Table 2:** Ten-Year Event Rates in Patients With and Without NSVT.

	NSVT (-)	NSVT (+)	Crude HR (95% CI)		HR (95% CI)		
Outcomes	n = 3547	n = 220		P value		P value	
All mortality	698(19.7)	78(35.5)	2.018 (1.597–2.550)	<0.001	1.362 (1.071–1.731)*	0.012	
*CV mortality*	*138(3*.*9)*	*18(8*.*1)*	*1*.*876(1*.*147–3*.*069)*	*0*.*012*	*1*.*444(1*.*076–1*.936)*	*0*.*014*	
All-cause hospitalization	1897(53.5)	145(65.9)	1.462 (1.235–1.732)	<0.001	1.175(0.990–1.395)**	0.066	
CV hospitalization	576(16.2)	62(28.2)	1.870 (1.43902.430)	<0.001	1.527(1.171–1.992)**	0.002	
Stroke	314(8.9)	36(16.4)	1.929 (1.366–2.724)	<0.001	1.436(1.014–2.032)**	0.041	
TIA	367(10.3)	42(19.0)	1.851 (1.338–2.559)	<0.001	1.483(1.069–2.057)**	0.018	
New-onset HF	326(9.2)	43(19.5)	2.299 (1.673–3.160)	<0.001	1.716(1.243–2.368)**	0.001	
**Subgroup of AF(-)**	n = 2987	n = 130					
New-onset AF	217(6.1)	20(9.9)	2.299 (1.673–3.160)	<0.001	1.428(0.914–2.231)**	0.118	

Values are number of events (%) unless otherwise indicated. Please see the S1 Table for crude HR for each parameter

AF indicate atrial fibrillation, CI, confidence interval; CV, cardiovascular; HF, heart failure; HR, hazard ratio; TIA, transient ischemic accident.

*HRs was adjusted for age, sex, hypertension, diabetes mellitus, chronic kidney disease, and anti-arrhythmic agents

** HRs was adjusted for age, sex, hypertension, diabetes mellitus, chronic kidney disease, anti-arrhythmic agents, and competing risk as mortality

### Risk factors

In this study, we used traditional markers to determine patient outcomes (age, sex, hypertension, diabetes mellitus, chronic kidney disease, anti-arrhythmia medication, and anti-hypertensive medication) as the old model. Adding NSVT as a new marker to estimate risk resulted in a significant improvement on the IDI (p < 0.01) for mortality, CV hospitalization, new-onset stroke, TIA, and new-onset HF.

### Subgroup analysis

**Fig 2:**
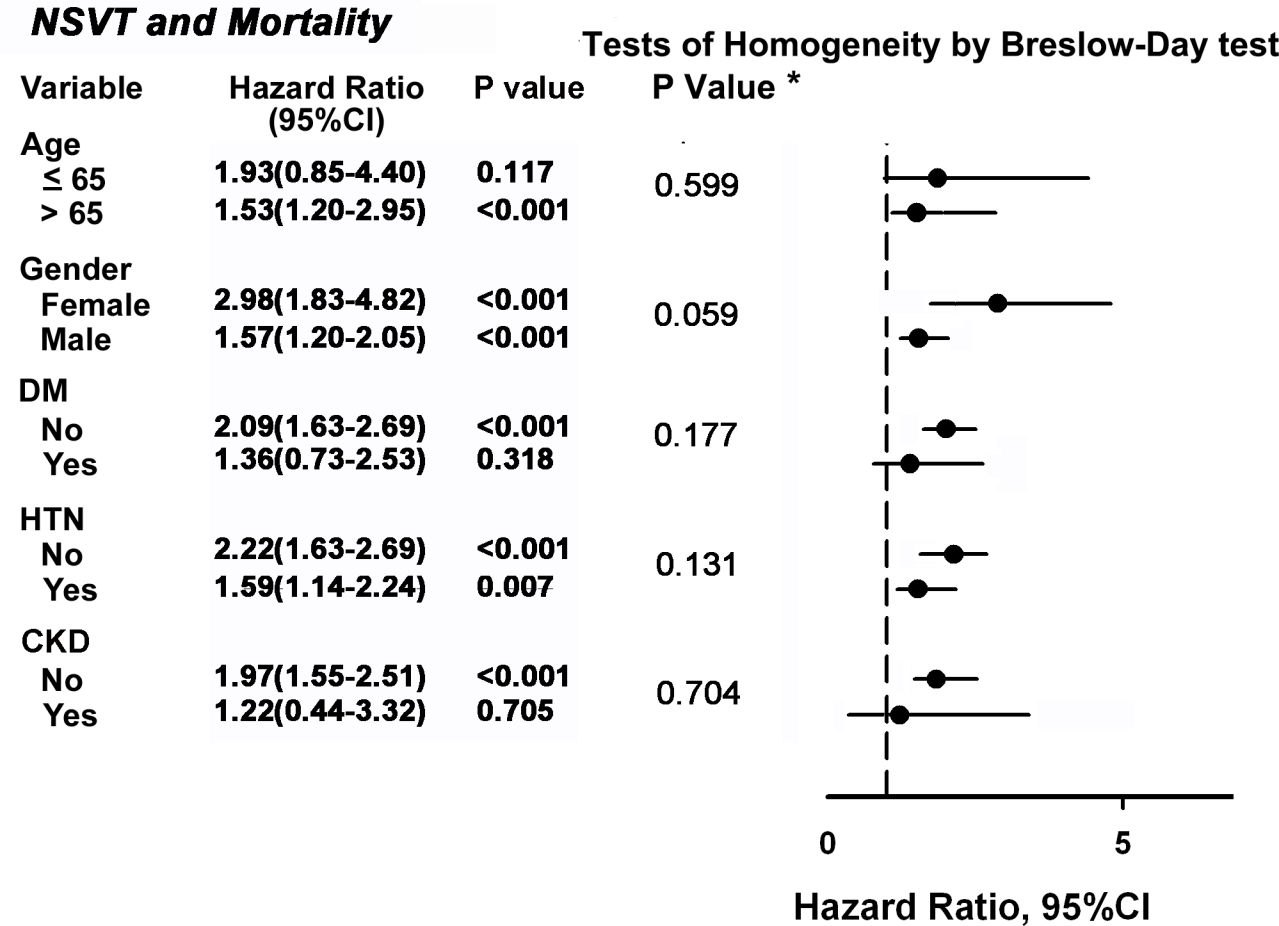
Forest plot for subgroup analysis for all-cause mortality. The hazard ratios of NSVT in comparison with no NSVT in different subgroups of patients with individual risk factors. CI indicates confident interval; CKD, chronic kidney disease; DM, diabetes mellitus; HTN, hypertension; NSVT, non-sustained ventricular tachycardia. *P value for the NSVT by each stratification variables interaction.

Patients with NSVT were at a higher risk of mortality in subgroup analyses of gender and hypertension (Fig 2). However, the higher risk of mortality in NSVT was not observed in patients under 65 years of age, with diabetes mellitus or with chronic kidney disease. Diabetes mellitus and chronic kidney disease may be associated with higher mortality by the disease itself, which diminished the influence of NSVT. Additionally, NSVT-related mortality was associated with death due to CV events, especially myocardial infarction and HF (Table 2 and S1 Fig).

## Discussion

### Main Finding

In this study, we demonstrated that NSVT in apparent structurally normal hearts was observed more frequently in older, sicker patients. After adjusting for these conditions, NSVT was associated with increased mortality, CV hospitalization, new-onset stroke/TIA, and development of systolic HF independent of sex, age, and other comorbidities. Furthermore, NSVT was associated with death due to CV events, especially myocardial infarction and HF.

### NSVT is Associated with Poor Clinical Outcome and Mortality

NSVT was associated with development of cardiac death attributed to HF and myocardial infarction and CV hospitalization in our study. Previous study has demonstrated that NSVT in apparent structurally normal hearts was associated with an increase in all-cause mortality and cardiovascular events,[2] which were compatible with our findings. Several earlier studies used exercise test, echocardiography and/or coronary arteriography to exclude structural heart disease and found that NSVT did not predict a coronary event.[19, 20] However, the subject population of these studies was limited. The results we obtained using our large study group suggest that NSVT is associated with poor clinical outcome. The increase in mortality also appeared to be partly explained by incident new-onset HF and ischemic stroke. NSVT was primarily related to myocardial ischemia or HF in a previous study.[6] One-third of HF patient deaths were complicated with unexpected sudden cardiac death during episodes of clinical worsening of HF.[21] NSVT might imply subclinical abnormality in apparently structural normal heart patients. Ventricular arrhythmia could be related to many reported non-cardiac systemic diseases, including metabolic, liver disease, electrolyte imbalance…etc. [22] NSVT in patients with apparent structural normal heart may have subclinical noncardiac disorder. Intensified follow-up and cardiac evaluation might be warranted for patients with NSVT, and future prospective trials may address this strategy.

Early separation of CV hospitalization curves was noticed. The reasons of CV hospitalization during first year were listed in S2 Table. No specific disease was found to be higher in NSVT patients compared to those without NSVT. NSVT might indicate a poor prognosis of underline disease.

### NSVT and HF

After adjusting for various confounding factors, we found that NSVT was associated with increments in the incidence rates of new-onset HF. NSVT might subsequently cause vagally-mediated late heart rate deceleration through the autonomic nervous system. [23, 24] Possible pathogenic mechanisms of this phenomenon include increased oxygen consumption and ventricular dyssynchrony.[25] Additionally, ventricular dyssynchrony asymmetrically increases wall thickness and alters blood flow in myocardium. [26] Furthermore, repeated dyssynchrony may impair calcium handling and contribute to contraction abnormality.

### NSVT and stroke

This study indicates that NSVT is an independent risk factor for incidence of stroke and TIA. An earlier study has found an association between ventricular ectopy and stroke development.[27] Another study demonstrated a decrease in the left atrial appendage flow velocity during ventricular pacing.[28] NSVT causes ventricular dyssynchrony and may contribute to mechanical abnormalities in the left atrial appendage, which might explain the potential for enhanced stroke/thromboembolic event. To the best of our knowledge, this study is the first investigation demonstrating that NSVT in the structural normal heart was a marker related to other stroke-causing diseases.

### Premature ventricular complexes (PVCs) burdens and morphologies

In several earlier studies, high burden PVCs and PVC morphology were associated clinical outcomes and an increase in the risk for more malignant dysrhythmias.[4, 11, 29] Our previous study demonstrated that patients with multiform PVC had an increased risk of mortality, hospitalization, new-onset HF, and new-onset AF independent of other clinical risks.[11]

PVCs and NSVT could be related to a tachycardia-induced cardiomyopathy in a healthy population.[29] However, the long-term prognosis has not been reported. In the present study, the mean PVC burden was 176 ± 423 beats per day and none of these patients had PVC burden more than 5% of the total beats. Therefore, the PVC burden was lower than that in previous studies. Furthermore, the incidence of multiform morphology PVCs between NSVT and non-NSVT groups was not different (37.1% vs 42.1%, P = 0.132)

### NSVT and clinical implication

This study had several strengths, including the large sample size, more extended follow-up period, and longer ECG recording. The presence of NSVT is documented by a standard 12-lead ECG used in a clinical setting. Twenty-four hour ambulatory ECG recording could provide higher detection rate of NSVT, which might offer an incremental value to help identify the high-risk group in patients with apparently normal hearts. Our study could not elucidate whether intervention with NSVT would reduce the adverse events because of the study design. However, our study highlighted the clinical importance of NSVT in patients with normal structure heart. Focused monitoring of this kind of patients is suggested to prevent adverse events.

## Study Limitation

There were some limitations to our study. First, the sicker patients were followed more frequently and had an increased likelihood of hospitalization with new onset of AF and HF. Second, patients were referred for Holter monitoring for various indications. NSVT could reflect the severity of underline diseases. Therefore patients with NSVT may be associated with early high CV hospitalization rate. Third, other unmeasured confounding factors might exist and increase the risks of the endpoints. Fourth, the patients’ characteristics were distinctly different between these two groups. Additionally, statistical methods might not completely adjust confounding factors. Further prospective randomized-control studies were required to evaluate the clinical implications. Fifth, the several secondary outcomes were relevant in our studies (ex. the new-onset HF and new onset stroke; the all-cause hospitalization and CV hospitalization). However, interactions among the different secondary endpoints could not be fully adjusted. Sixth, the burden of ventricular ectopy is low at enrollment. Further prospective study with serial 24-hour Holter follow-up is warrant.

## Conclusions

NSVT in patients with apparent structurally normal hearts was independently associated with a higher risk of mortality, CV hospitalization, stroke/TIA, and new-onset HF in this 10-year follow-up study.

## Supporting Information

S1 Fig Cause of death. Presence of NSVT was associated with death due to CV events, including heart failure and myocardial infarction. CV indicates cardiovascular; HF, heart failure; MI, myocardial infarction; SCD, sudden cardiac death.
(TIF)

S1 Table Crude hazard ratio for long-term outcome. (DOCX)

S2 Table CV hospitalization during the first year. (DOCX)

## Abbreviations

AF: atrial fibrillation
CCHIA: Collaboration Center of Health Information Application
CI: confidence interval
CV: cardiovascular
ECG: electrocardiography
HF: heart failure
HR: hazard ratio
IDI: integrated discrimination improvement
NSVT: non-sustained ventricular tachycardia
NHIRD: National Health Insurance Research Database
PVCs: premature ventricular complexes
TIA: transient ischemic accident
