# Supplementary figures and images for: Long-Term Outcome of Non-Sustained Ventricular Tachycardia in Structurally Normal Hearts
Source: PLoS One. 2016 Aug 22;11(8):e0160181. doi: 10.1371/journal.pone.0160181 (PMC4993359; doi:10.1371/journal.pone.0160181)

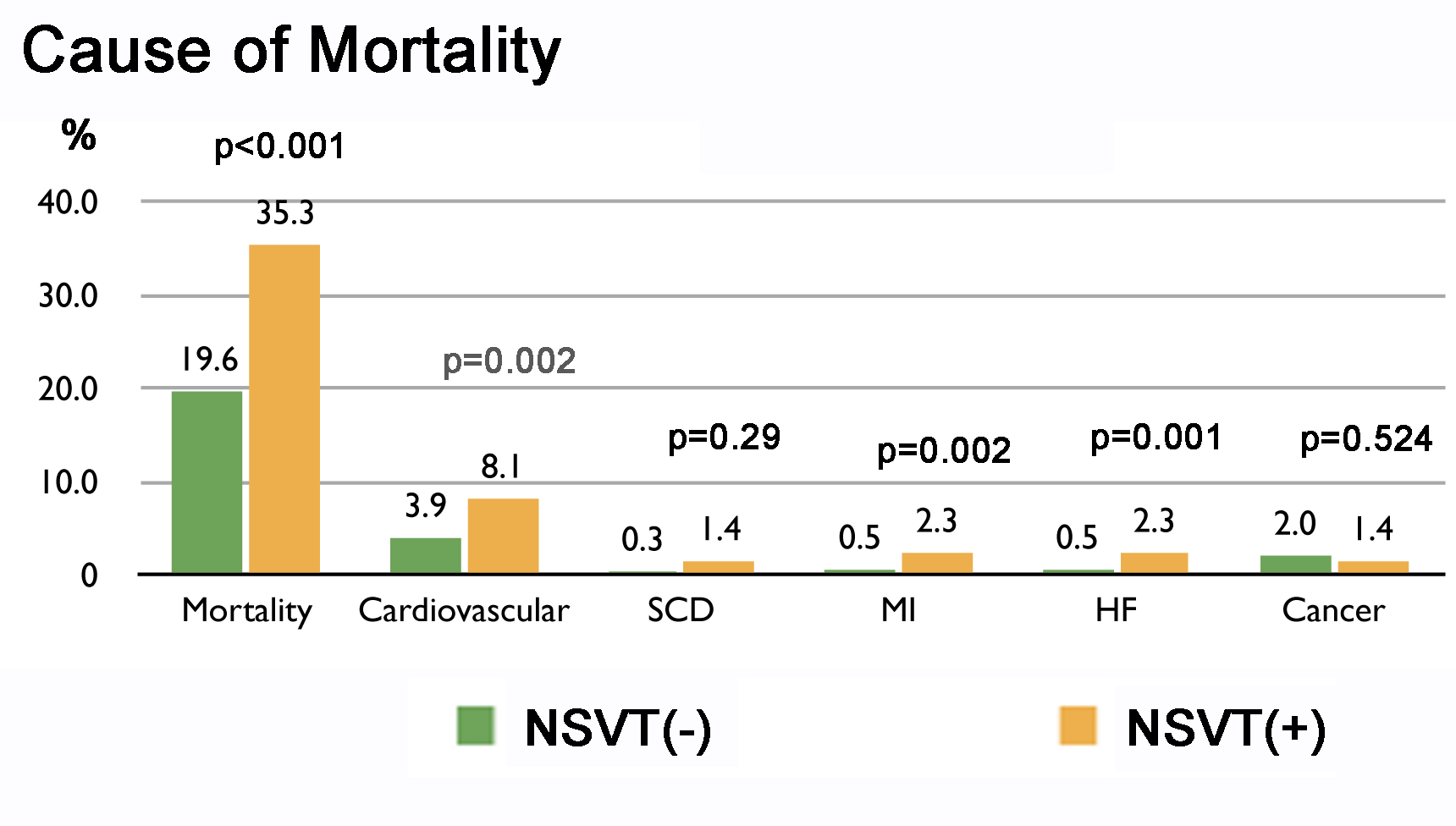

Supplement: S1 Fig — Presence of NSVT was associated with death due to CV events, including heart failure and myocardial infarction. CV indicates cardiovascular; HF, heart failure; MI, myocardial infarction; SCD, sudden cardiac death. (TIF) [file pone.0160181.s001.tif]
