# Supplementary material for: Long-Term Outcome of Non-Sustained Ventricular Tachycardia in Structurally Normal Hearts
Source: PLoS One. 2016 Aug 22;11(8):e0160181. doi: 10.1371/journal.pone.0160181 (PMC4993359; doi:10.1371/journal.pone.0160181)
Supplement: S1 Table — (DOCX) [file pone.0160181.s002.docx]

| Supplemental Table 1. Crude Hazard Ratio for Long-Term Outcome | | | |  |
| --- | --- | --- | --- | --- |
| **Outcome** | **Crude HR (95% CI)** | **P Value** |  |  |
| **All mortality** |  |  |  |  |
| NSVT | 2.018 (1.597-2.550) | <0.001 |  |  |
| Age (years) | 1.084 (1.073-1.096) | <0.001 |  |  |
| Male | 1.556 (1.323-1.831) | <0.001 |  |  |
| Hypertension | 1.434 (1.164-1.766) | <0.001 |  |  |
| Diabetes mellitus | 1.044 (0.876-1.212) | 0.567 |  |  |
| Chronic kidney disease | 3.713 (2.374-5.807) | <0.001 |  |  |
|  |  |  |  |  |
| All-cause Hospitalization |  |  |  |  |
| NSVT | 1.462 (1.235-1.732) | <0.001 |  |  |
| Age (years) | 1.025 (1.023-1.028) | <0.001 |  |  |
| Male | 1.464 (1.338-1.602) | <0.001 |  |  |
| Hypertension | 1.514 (1.384-1.656) | <0.001 |  |  |
| Diabetes mellitus | 1.437 (1.241-1.663) | <0.001 |  |  |
| Chronic kidney disease | 2.144 (1.477-3.114) | <0.001 |  |  |
|  |  |  |  |  |
| CV Hospitalization |  |  |  |  |
| NSVT | 1.870 (1.43902.430) | <0.001 |  |  |
| Age (years) | 1.026 (1.021-1.031) | <0.001 |  |  |
| Male | 1.808 (1.527-2.140) | <0.001 |  |  |
| Hypertension | 1.680 (1.436-1.966) | <0.001 |  |  |
| Diabetes mellitus | 2.028 (1.619-2.540) | <0.001 |  |  |
| Chronic kidney disease | 1.645 (0.852-3.176) | 0.138 |  |  |
|  |  |  |  |  |
| New-onset Stroke |  |  |  |  |
| NSVT | 1.929 (1.366-2.724) | <0.001 |  |  |
| Age (years) | 1.049 (1.041-1.057) | <0.001 |  |  |
| Male | 2.052 (1.623-2.595) | <0.001 |  |  |
| Hypertension | 2.079 (1.686-2.565) | <0.001 |  |  |
| Diabetes mellitus | 2.018 (1.492-2.730) | <0.001 |  |  |
| Chronic kidney disease | 0.288 (0.040-2.052) | 0.214 |  |  |
|  |  |  |  |  |
| New-onset TIA |  |  |  |  |
| NSVT | 1.851 (1.338-2.559) | <0.001 |  |  |
| Age (years) | 1.030 (1.024-1.037) | <0.001 |  |  |
| Male | 2.249 (1.801-2.808) | <0.001 |  |  |
| Hypertension | 1.621 (1.331-1.974) | <0.001 |  |  |
| Diabetes mellitus | 1.611 (1.192-2.177) | 0.002 |  |  |
| Chronic kidney disease | 1.057 (0.395-2.830) | 0.912 |  |  |
|  |  |  |  |  |
| New-onset Heart Failure |  |  |  |  |
| NSVT | 2.299 (1.673-3.160) | <0.001 |  |  |
| Age (years) | 1.061 (1.052-1.070) | <0.001 |  |  |
| Male | 1.921 (1.533-2.407) | <0.001 |  |  |
| Hypertension | 1.856 (1.511-2.278) | <0.001 |  |  |
| Diabetes mellitus | 2.619 (1.997-3.435) | <0.001 |  |  |
| Chronic kidney disease | 2.664 (1.322-5.367) | 0.006 |  |  |
|  |  |  |  |  |
| New-onset Atrial fibrillation |  |  |  |  |
| NSVT | 1.898 (1.219-2.956) | 0.005 |  |  |
| Age (years) | 1.027 (1.019-1.035) | <0.001 |  |  |
| Male | 1.817 (1.371-2.407) | <0.001 |  |  |
| Hypertension | 1.683 (1.294-2.189) | <0.001 |  |  |
| Diabetes mellitus | 1.829 (1.245-2.685) | 0.002 |  |  |
| Chronic kidney disease | 1.840 (0.685-4.947) | 0.227 |  |  |
| CI indicates confidence interval; CV, cardiovascular; HR, hazard ratio; NSVT, non-sustained ventricular tachycardia; TIA, transient ischemic accident. | | | |  |
