# Supplementary material for: Long-Term Outcome of Non-Sustained Ventricular Tachycardia in Structurally Normal Hearts
Source: PLoS One. 2016 Aug 22;11(8):e0160181. doi: 10.1371/journal.pone.0160181 (PMC4993359; doi:10.1371/journal.pone.0160181)
Supplement: S2 Table — (DOCX) [file pone.0160181.s003.docx]

| Supplemental Table 2. CV hospitalization during the first year | | | |
| --- | --- | --- | --- |
|  | NSVT (+)  N=220 | NSVT(-)  N=3547 | P value |
| CV hospitalization | N=26 (11.8%) | N=226 (6.4%) | 0.003 |
| Heart Failure | 6 (2.7%) | 45 (1.3%) | 0.120 |
| Atrial fibrillation | 4 (1.8%) | 38 (1.1%) | 0.307 |
| Infection | 3 (1.4%) | 24 (0.7%) | 0.207 |
| Near-syncope or syncope | 2 (0.9%) | 26 (0.7%) | 0.677 |
| Cerebral vascular accident | 3 (1.4%) | 29 (0.8%) | 0.431 |
| Hypertension | 4 (1.8%) | 39 (1.1%) | 0.207 |
| Pericardial effusion | 0 (0.0%) | 2 (0.1%) | 0.999 |
| Sudden cardiac arrest | 1 (0.1%) | 1 (0.03%) | 0.113 |
| Chest pain | 0 (0.0%) | 2 (0.1%) | 0.999 |
| Aortic aneurysm | 1 (0.5%) | 5 (0.1%) | 0.303 |
| Deep vein thrombosis | 1 (0.1%) | 7 (0.2%) | 0.382 |
| Bradycardia | 1 (0.5%) | 8 (0.2%) | 0.419 |
| CV indicates cardiovascular | | | |
